# Neuroinflammation Following Traumatic Brain Injury: Take It Seriously or Not

**DOI:** 10.3389/fimmu.2022.855701

**Published:** 2022-03-22

**Authors:** Rui-zhe Zheng, Kuin-yu Lee, Zeng-xin Qi, Zhe Wang, Ze-yu Xu, Xue-hai Wu, Ying Mao

**Affiliations:** ^1^ Department of Neurosurgery, Huashan Hospital, Shanghai Medical College, Fudan University, Shanghai, China; ^2^ National Center for Neurological Disorders, Shanghai, China; ^3^ Shanghai Key Laboratory of Brain Function and Restoration and Neural Regeneration, Shanghai, China; ^4^ Neurosurgical Institute of Fudan University, Shanghai, China; ^5^ Shanghai Clinical Medical Center of Neurosurgery, Shanghai, China; ^6^ State Key Laboratory of Medical Neurobiology and Ministry of Education (MOE) Frontiers Center for Brain Science, School of Basic Medical Sciences and Institutes of Brain Science, Fudan University, Shanghai, China; ^7^ Department of Integrative Medicine and Neurobiology, Institute of Integrative Medicine of Fudan University Institute of Brain Science, School of Basic Medical Sciences, Fudan University, Shanghai, China

**Keywords:** neuroinflammation, traumatic brain injury, advance, bow-tie framework, take seriously

## Abstract

Traumatic brain injury (TBI) is associated with high mortality and disability, with a substantial socioeconomic burden. With the standardization of the treatment process, there is increasing interest in the role that the secondary insult of TBI plays in outcome heterogeneity. The secondary insult is neither detrimental nor beneficial in an absolute sense, among which the inflammatory response was a complex cascade of events and can thus be regarded as a double-edged sword. Therefore, clinicians should take the generation and balance of neuroinflammation following TBI seriously. In this review, we summarize the current human and animal model studies of neuroinflammation and provide a better understanding of the inflammatory response in the different stages of TBI. In particular, advances in neuroinflammation using proteomic and transcriptomic techniques have enabled us to identify a functional specific delineation of the immune cell in TBI patients. Based on recent advances in our understanding of immune cell activation, we present the difference between diffuse axonal injury and focal brain injury. In addition, we give a figurative profiling of the general paradigm in the pre- and post-injury inflammatory settings employing a bow-tie framework.

## Background

With an incidence of more than 50 million people per year and a leading cause of death and disability, traumatic brain injury (TBI) brings a substantial social and economic burden worldwide (1). There are two distinct subtypes of TBI: one is focal brain injury (FBI), which is caused by the brain hitting against the cranium; the other is diffuse axonal injury (DAI) (2), which results from a rotational motion that leads to brain stretching and tearing. TBI is a complex event that refers to any insult to the brain, resulting in primary (direct) mechanic injury and secondary (indirect) insult to the brain parenchyma (3). It remains challenging to fully reveal the pathologically heterogeneity following TBI as it is linked to excitotoxicity, neuroinflammation and cytokine damage, and oxidative damage (4).

On account of the inflammation and white matter (WM) degeneration that will persist for many years after just one single TBI (5), the role of neuroinflammation following TBI has come into focus in recent studies (6, 7). To our knowledge, immune activation can be produced both by endogenous brain cells and circulating inflammatory cells, and together, they are driven by a variety of cytokines and chemokines (8). It is a sterile immune response for the absence of pathogen infection, and many endogenous damage-associated molecular pattern molecules (DAMPs) are generated in the processes. Moreover, the purinergic signaling [including P1 receptors in response to adenosine and P2 receptors in response to adenosine triphosphate (ATP) or adenosine diphosphate (ADP)] will provide energy for immune activation (9). Collectively, it can quickly trigger an inflammatory cascade of the local signaling in neurons, glia (microglia and astrocytes), and the exogenous signal in peripheral recruit cells (monocyte, macrophages, neutrophils, and T cells) within minutes following TBI (10). In addition, the microglia phenotypes are highly plastic since they depend on the different microenvironmental settings of TBI (e.g., M1-like will increase uncontrolled neuroinflammation, and M2-like is more likely associated with an anti-inflammatory effect) (11). The astrocytes can provide support and nutrition to homeostasis and play an essential role in glial scar formation that obstructs axon regeneration (12). In short, the neuroimmune response following TBI is often complicated, which can be summarized as 1) more than one single cell and pathway, and 2) a simple classification of a cytokine or cell as detrimental or beneficial may not be appropriate.

Applying the graph theory in interpretation was proven to be a powerful way to reveal the regularity of biological evolution. Significantly, a bow-tie framework (13) can figuratively integrate the concepts of degeneracy and pluripotency in the dynamic, organized types. Generally speaking, we can use its overall structure to display both a highly fluctuating or “sloppy” program and a high concentration or “tidy” environment. In the past, a bow-tie framework was used for grasping the immune system’s complexity, which offers a way to understand the common principle of the metabolic process, protein interactome, and gene circuits (14, 15). Along with the development of transcriptomic and proteomic profiling, immune cell studies have moved from the simplistic microglial M1/M2 classification scheme to the specific delineation of innate immune functions (7). Therefore, it is practicable to present a panoramic view of the immunological events that follow TBI and describe the dynamic aspects of neuroinflammation.

This review will briefly present the neuroinflammation in TBI and summarize the relative difference in DAI. We point out the available advances of TBI-related neuroinflammation in current human and animal model studies. We also highlight the challenge of putting immunotherapies for altering clinical outcomes in patients with TBI.

## Immunogenicity in the Central Nervous System

In the central nervous system (CNS), the critical role of resident microglia has been receiving increasing concern as it can be either pro-inflammatory or anti-inflammatory (16). The different immunological phenotypes of microglia reflect their contradictory functions in maintaining homeostasis and their contribution to TBI recovery or aiding injury (17). Meanwhile, the inflammatory response is equally essential in astrocytes following TBI; it plays a critical role in the energy metabolism supply and blood–brain barrier (BBB) injury and can produce neuroprotective factors or cytotoxic mediators. As far as we know, the glial responses following TBI are not isolated but coordinated and integrated, and the crosstalk between microglial activations and astrocytic responses has been widely studied (18). In response to TBI, the activation of microglia will promote astrogliosis and persistent inflammation, and the astrocytes will communicate with it through the cytokines or chemokines they release. In addition, the infiltrating immune subsets that were recruited from the circulation and accumulated in the brain are indispensable for neuroinflammation in TBI (19). It has been found that those subsets include CNS-associated macrophages (CAMs), various types of monocytes (Ly6Clo or Ly6Chi types, monocyte-derived cells), classical or plasmacytic dendritic cells (cDCs or pDCs), T cells, B cells, and natural killer (NK) cells (20). However, there are insufficient investigations about the timing and spatial activation level, as well as the crosstalk between CNS immune cells and peripheral invasive subsets.

It has recently been found that meningeal lymphatic vessels (mLVs) are involved in CSF clearance (21). To the best of our knowledge, mLVs could alter the accessibility of immune neuromodulators carried by CSF-borne to the brain parenchyma, thereby changing their effects on the immunogenicity of CSF (22). An interesting phenomenon is it can be dysfunctional with the occurrence of TBI, of which the increased intracranial pressure plays a crucial role. The rejuvenation of mLVs’ drainage function in an animal model can ameliorate TBI-induced pathogenesis (23). Although the BBB dysfunction and mLVs can be regarded as the paths of the intra- and extracranial immune component exchange, the exact one they take and priority of each route remain controversial.

Significantly, the advanced proteomic profiling technologies enable us to catch the phenotype information of neuroinflammation in different TBI phases; the high-throughput single-cell sequencing (scRNA-seq) technologies allow us to identify homeostatic or disease-specific myeloid subsets (24). For example, gene profiling and pathway analyses enable us to distinguish invading peripheral monocytes from resident microglia (25); scRNA-seq reveals distinct inflammation-induced microglia signatures under inflammatory conditions (26); scRNA-seq gave a comprehensive transcriptional and translational resource to explain the differences of neuroinflammation caused by gender difference (27). Moreover, a recent study found that the CD4 T cell in a healthy brain was distinct from those in the circulation, and the resident microglia will suspend in the absence of CD4 T cells (28). At this point, it can accurately distinguish the brain-resident immune cells from the infiltrating immune cells in patients with TBI.

Collectively, the immunogenicity of CNS is composed of innate immunity and peripheral infiltrating immune cells. Recent evidence has shown that TBI will raise the phenotypic difference between microglia, macrophages, and DC, and the infiltrating subsets will alter the microglia activation. To date, the crosstalk between microglia and circulating immune cells; the role of cytokines, chemokines, and signaling pathways in the activation process; and the functions of regulators in the M1/M2 polarization have made the aspects of neuroinflammation induced by TBI particularly topical (29–31). Regardless of the complexity of CNS immunogenicity, it is practical to establish the regularity of neuroinflammation through the synopsis of some critical mediators following TBI.

## Triggering Signal

### Initiators

As an initial signaling mediator, DAMPs are regulated by the pattern recognition molecules such as toll-like receptors (TLRs), nucleotide-binding oligomerization domain-like receptors (NLRs), scavenger receptors, and the purinergic system to initiate the inflammatory cascade (32). The most critical transmembrane regulators, TLRs, were observed in neurons, microglia, astrocytes, and oligodendrocytes. Experimental studies have shown that the TLR2 and TLR4 will be upregulated more than two-fold at 24 h, peak at 7 days, and decline at 14 days in the peripheral lesion (33). The primary cellular sources of TLRs are the microglia in injured cortex areas and astrocytes in subcortical WM (34). Based on the adaptor protein recruited by TLRs, the TLR-related signaling pathways can be classified as a myeloid differentiation primary response gene 88 (MyD88)-dependent pathway and MyD88-independent pathway. The expression of MyD88 protein will increase from 6 h to 7 days after TBI, peaking on the third day, parallel to the increase of Nuclear transcription factor (NF-kB) and pro-inflammatory cytokines. To our knowledge, MyD88 was colocalized with microglia in the injured areas and with astrocytes in subcortical WM (33). Moreover, the expression of NF-kB will peak at 7 days and then begin to decrease at 14 days after TBI. TLR4 and transforming growth factor-b-activated kinase 1 expression increased significantly after TBI. Meanwhile, the function of TLR4 in the TBI process has been examined in the previous controlled cortical impact (CCI) model, of which TLR4-deficient mice had smaller brain lesions than wild-type controls (35). Recent studies have shown that inhibiting the TLR4 signaling pathway will attenuate the inflammation through regulating the microglial M1/M2 phenotype (36). Therefore, it is necessary to evaluate the distribution of TLRs and the role of downstream signals as they may be associated with the repair and regeneration of the CNS. However, the time paradigm of different TLR expressions is worthy of a detailed investigation to determine whether early activation is beneficial while later activation is detrimental (37).

### Inflammasomes

Upon the recognition of DAMPs, the macromolecular complex, referred to as an inflammasome, includes NLR (e.g., NLRP3, NLRP1) and non-NLR proteins [e.g., absent in melanoma 2 (AIM2)]. NLRs were cytosolic receptors for DAMPs, and they can promote the cleavage of pro-caspase-1 into its active form (caspase-1) *via* interactions with caspase activation and recruitment domains (CARDs) located within the inflammasome or in association with an apoptosis-associated speck-like protein containing a CARD (ASC) (7). The activation of caspase-1 will mediate the interleukin (IL)-1b and IL-18 secretion, which are excellent diagnostic and predictive biomarkers of TBI (38). Inflammasomes can assemble in macrophages, microglia, or astrocytes, and their activation form can generate pro-inflammatory or anti-inflammatory cytokines. NLRP3 inflammasome has been widely studied as it plays a crucial role in regulating cerebral edema and secondary inflammation (39). Interestingly, a previous study found that the heightened levels of NLRP1, ASC, and caspase-1 detected in the CSF are correlated with a more unfavorable neurological outcome in patients with moderate and severe TBI (40). Further studies have indicated that the NLRs and AIM2 inflammasome-mediated pyroptosis could aggravate BBB damage (41), and the pyroptosis process can be attenuated *via* the HMGB1/TLR4/NF-κB pathway (42). However, a recent CCI mice study suggested an irrelevant role of the NLRP1 and ASC inflammasome on histopathology and motor recovery (43). At this point, further studies are required to explore whether NLRP1 and/or NLRP3 inflammasome activation will uniformly alter the pathogenicity in TBI.

### Endogenous Proteins

Meanwhile, multiple endogenous proteins such as high mobility group box one protein (HMGB1) and heat shock proteins (HSPs) will upregulate in neural and inflammatory cells following TBI. HMGB1, a cytokine released by glia and neurons upon inflammasome activation and that activates Toll 4 and the receptor for advanced glycation end products (RAGE). The HMGB1-RAGE interaction that mediated HMGB1 endocytosis followed by direct NF-κB activation has been widely studied, while the HMGB1 interacts with MD-2 to trigger TLR-4, and downstream signaling was relatively rare (36, 44). Despite the fact that the HMGB1 blockade can dampen neuroinflammation post-TBI, the effects of HMGB1 antagonism on neurogenesis remain been elucidated. Acetylated HMGB1 represents the active release of immune cells, while non-acetylated HMGB1 indicates a passive release from necrotic cells. Indeed, HMGB1 is passively released following TBI, which results in an activated microglia phenotype and facilitates increased BBB permeability *via* increased AQP4 expression in astrocytes (45). Recent experimental evidence has demonstrated the positive effects of HMGB1 antagonism on the pathological and behavioral parameters following TBI. However, some fundamental problems such as elucidating isoform, receptor, cell, and clinical impact require further investigation (46). HSPs, the protein chaperones induced by TBI, are known to participate in the TLR/NLR signaling pathway and serve as TLR ligands. Importantly, HSPs can present pro- and anti-inflammatory effects and stimulate immune responses by activating antigen presentation. Previous studies found that HSP70 significantly increases the lesion size, increasing the expression of metalloproteinases with worsened behavior (47). Further studies found that it provides neuroprotection *via* anti-inflammatory effects by the HSP70/NF-κB/IL-6/synapsin I axis in the injured brains and also found that TBI will lead to different types of transcriptional activation or inhibition (48). However, it remains to be seen whether there is a dynamic or temporal relationship in the HSP70-mediated pro- or anti-inflammatory processes after TBI.

### Purinergic Receptors

The purinergic receptors play a pivotal role in neuroinflammation responses as it serves as an alarmin that induces extracellular ATP release (49). The initial evidence was that purinergic receptor signaling participates in the activation and morphological transformation of microglia. A subsequent cell interaction study found that microglia transform astrocytes into a neuroprotective phenotype *via* the downregulation of the P2Y1 purinergic receptor (50, 51). It is known that the P2X7 receptor, an gated ion channel activated by ATP, can induce BBB disruption and the chemotaxis of peripheral immune cells to the CNS (52). For example, it can promote neutrophil recruitment to the damaged brain parenchyma within 1–3 ho following TBI. Moreover, it has been found that the antagonists and immune inhibitors of P2X7 receptors can reduce the number of apoptotic neuron deaths and increase the survival of the neurons in the injured and adjacent regions (53). A recent CCI study reported that the pharmacological inhibition of Pannexin-1 (Panx1), an essential conduit for ATP, will markedly reduce immune cell infiltration and BBB leakage (54). Those data demonstrate that the responses mediated by purinergic receptors are diverse and are influenced by the receptor expression pattern, nature and timepoint of the injury, and type of immune cell activation. In addition to the above-mentioned signaling pathway, purinergic signaling can also contribute to the activation of inflammasomes. Although it was less reported in the TBI, it has been confirmed that the activation of the P2X7 receptor contributes to the activation and proliferation of NLRP3 (55).

### Molecule Expression

To our knowledge, the expression patterns of inflammatory genes in parenchymal and non-parenchymal injured tissues are qualitatively similar. The genes associated with cytokines, chemokines, glial activation, antigen presentation, and phagocytosis, among others, can be upregulated and/or deregulated after TBI. It is noteworthy that the fastest restoration to the baseline expression level will take at least 10 days, so repeated measurements are required during this period to obtain an accurate diagnosis (56). In a comparing study, more than 89% of CCI differentially expressed mRNAs were observed, including changes in inflammatory genes, such as macrophage inflammatory protein (MIP)-1α (CCL3), CXCL1, IL-1α, IL-1β, and IL-6 (57). Nowadays, significant advances in identifying a molecule expression program that guides the ensuing immune response following TBI comes from the application of scRNA-seq and transcriptome sequencing technology. A recent study revealed that CNS-resident macrophages could quickly generate context-dependent subsets, guided by the dynamics of molecule expression during neuroinflammation (19). Another study found that a deficient C-C chemokine receptor-2 (Ccr2) after TBI will reduce the expression of the IFN-responsive gene (IRF7), which will shape our understanding in finding the targets from the diversity and crosstalk of neuroinflammation (30). Therefore, permanent changes in the CNS molecular expression may explain the immune heterogeneity observed in patients with TBI.

### Complement Activation

The complement system could not be neglected as it can present either deleterious or neuroprotective effects in TBI. It can be activated *via* three different pathways: the classical, alternative, and mannose-binding lectin (MBL) pathways. Recently, many studies have found that neurons and glial cells can synthesize complements; the mRNA-encoding receptors of C3a and C5a are widely expressed in the CNS (58). Initial immunohistochemistry studies found that C1q, C3b, C3d, and the membrane attack complex (MAC) were elevated in clinical patients and animal models after TBI. Subsequent shreds of evidence suggested that C3 inhibition was associated with reduced peripheral neutrophil and leukocyte infiltration, and C5 inhibition was related to the reduction in leukocyte infiltration and edema in the vicinity of the lesion, and increasing the MAC attachment to cell membranes will improve neuronal loss and worse neurological outcomes (59, 60). Similarly, a reduced microglial activation and neuronal death can be obtained when administrating a C5 complement inhibitor or a C6 antisense oligonucleotide (61, 62). In addition, there has been evidence that reactive microglia activate the complement by the local synthesis of MAC/C5b-9 complex, which involves the activation of the complement cascade (63). There has also been supporting evidence that the complement-mediated microglial phagocytosis of synapses provides the pathological relationship between acute injury and chronic neurodegeneration (64).

## Microglia Activation

As the first responders to TBI, microglia will be activated rapidly and change their morphology to form larger cell bodies with ramified cellular structures (65). After activation, microglia can proliferate and migrate to the injury location and polarize and induce the release of cytokines (66). This process is mediated by purinergic receptors (P2Y6, P2X4, and P2Y12) and Tyro3, Axl, and myeloid-epithelial-reproductive (Mer) (TAM) receptor tyrosine kinases (31). Microglia proliferation starts within 24 h and can continue for several weeks (67). Although the microglial polarization remains uncertain, the M1 and M2 phenotypes are the two widely used terminologies of activated microglia (68). The M1 phenotype majorly secretes pro-inflammatory cytokines (e.g., IFN-γ, TNF-α, and IL-1β) and chemokines (e.g., CCL-2, CCR-2, and CXCL-1), while M2 phenotype will be involved in the release of anti-inflammation cytokines for CNS repair (69). Indeed, microglial M1/M2 always plays a mixable role in pro- and anti-inflammatory responses; the M1 phenotype can secrete the anti-inflammatory cytokine IL-10, and the M2 phenotype presents an anti-inflammatory effect (70). If the M2 phenotype becomes overwhelmed and the M1 phenotype activity increases during the insult, chronic neuroinflammation and long-term damage will occur continuously (6). At this point, the redirection of microglia polarization to the M2 phenotype seems to be an excellent path to TBI immunotherapy for their neuroprotective contribution. For example, the peroxisome proliferator-activated receptor γ (PPAR-γ) pathway (71) and TLR4 signaling pathway (72, 73) have been proven to be related to the attenuation of inflammation by promoting the polarization of beneficial M2 microglia. However, the spatial phenotype is still challenging for current microglial studies because it can rapidly change their morphology according to the dynamic microenvironment.

Inflammatory gene studies that profile microglia gene expression at the single-cell level is becoming the current hot topic. In an scRNA-seq study, a time-dependent and injury-associated change in microglial gene expression networks has been found, of which a biphasic pattern of IL-4, IL-10, and interferon-gamma (IFN-γ) gene expression changes between 14 and 60 days post-injury (74). It has been found that microglia exhibited distinct clustering with an increased IFN-1 and neurodegenerative or damage-related genes at 7 days post-TBI; it was a critical time point in the transition from acute to chronic pathogenesis (18, 75). Meanwhile, gene manipulation techniques such as the Cre-Lox approach and the microglia-specific promoters would also expand our understanding of the role of microglial genes following TBI. Recently, there is strong evidence that the cell-specific knockout of p38α in microglia will significantly reduce the production of pro-inflammatory cytokines or chemokines and the recruitment of monocytes into the brain (76). Similarly, the overexpression of the charged multi-vesicular body protein 4b (CHMP4B) can relieve the microglial necroptosis (77). Therefore, further conditional and inducible gene manipulation experimentation could be carried out to investigate the microglia that contribute to TBI-related neuroinflammation.

In addition, microglia-derived extracellular vesicles (EVs) are suggested to be involved in neuroinflammation and cell communication following TBI (78). It has a well-defined lipid bilayer with the surface markers of the reflection of the cell origination and an aqueous core including the cytokines, growth factors, and microRNAs (miRNAs) (79). There are 49 unique proteins in the EV released from activated microglia, and they can carry a variety of molecular constituents such as miRNAs (80). For example, microparticles (MPs), a member of the EV family loaded with pro-inflammatory molecules, could independently initiate inflammatory responses in the injured brain and stimulate systemic immune responses (81). It has been reported that increased miR-124-3p in microglial exosomes can inhibit neuroinflammation and contribute to neurogenesis (82), and EVs can carry anti-inflammatory molecules for reducing neuroinflammation following TBI (83). Moreover, miRNA-21-5p are highly enriched in neuron-derived exosomes after M1 microglial polarization following TBI. Evidence had shown that the localization of miRNA-21-5p increased in the lesion as the M1 microglia gathering and cyclic cumulative damage between neurons and microglia was caused by miR-21-5p and exosomes. Similarly, exosomes with miRNA-124-3p are released by microglia, which can present anti-inflammatory and neuroprotective effects through M2 microglial activation (84). At this point, the microglia can be manipulated to form a pro-regenerative or neuroprotective phenotype following TBI (85).

## Peripheral Immune Cell Infiltration

Neutrophils are often among the first peripheral immune cells infiltrating into contused brain tissue within a few hours following TBI, directed by the purines, cytokines (e.g., TNF-a and IL-1b), and neutrophil chemoattractant molecules (e.g., CXCL1, CXCL2, and CXCL3) (7). Combined with the expression of adhesion molecules (ICAM-1), neutrophils can accelerate the migration through BBB and the brain parenchyma (86). The contribution of infiltrated neutrophils to TBI pathogenesis varies as follows: 1) neutrophil depletion can reduce edema and microglial activation (87); 2) similar results can be obtained in the CCL2 genetic deficient model (88), and 3) the neutrophil extracellular traps (NETs) can improve neurological function (89). Collectively, the available changing of those pathogeneses might be related to oxidative signaling [e.g., reactive oxygen species (ROS)], as it often alters the expression of the NADPH and iNOS enzymes in different TBI courses (90).

After the neutrophil recruitment, it is often followed by the arrival of monocytes, then converted into macrophages in the injured brain. It occurs within 1–2 days for monocytes entering the CSF and parenchyma and remains there for weeks after TBI (91). Hereto, the widely accepted mechanism of monocyte recruitment following TBI was the local production of the chemokine CCL2 in CSF (92). Macrophages can be either beneficial or detrimental, depending on their functional properties. It is worth noting that the phenotypic differentiation between peripheral macrophages and the resident microglia is age-dependent, so they may be an available aggregation unit rather than a different entity (93). Although it is difficult to distinguish the microglia and macrophages, flow cytometry has recently made it possible to discriminate between the two cell types. A comparative study found that ROS were more obvious in macrophages rather than microglia, and the NADPH-2, IL-1β, and CD68 were higher in macrophages, whereas the TGF-β1, IL-6, and TNF-α are higher in microglia (94). In this regard, macrophages can be regarded as the aggravating cell type, while the activated microglia may play a favorable role during the acute phase of TBI. The latest advancement is a study that identifies the beneficial effect derived from a combination of the macrophage colony-stimulating factor (M-CSF), IL-6, and transforming growth factor-β (TGF-β), termed M6T, especially the cosecretion of microglia, macrophages, and endothelial cells (95).

T cells infiltrating into the damaged brain parenchyma can be observed in the CCI model posttraumatic 3–5 days, and a prominent infiltration was confined to the perilesional cortex and hippocampus. To be more specific, most parenchymal T cells were resident memory CD8+ T cells, which may be mediated by mLVs (96). It has been found that the depletion of regulatory T cells (Tregs) can increase T-cell infiltration reactive gliosis and IFN-γ gene expression (97). Despite scarce evidence existing, the pharmacological depletion of CD8+ T cells may produce a neuroprotective effect *via* a Th2/Th17 immunological shift (98). It will draw our attention to the specific subsets of infiltrating T cells and even B cells that affect the neurological dysfunction or neuroimmune response.

## Glial Interaction

It has been found that the microglial activation following TBI could promote astrogliosis and persistent neuroinflammation, and the ATP releasing from purinergic receptors on astrocytes that lead to microglial recruitment has been generally considered a communication signal (99). Although purinergic receptors seem to be pivotal mediators, astrocytes and microglia can communicate through the cytokines and extracellular mediators (exosomes) they release. Previous studies found that the HMGB1 released by neurons will activate TLR4 in microglia and subsequently release IL-6, which, in turn, activates astrocytes to increase the AQP4 expression, which eventually causes astrocyte swelling and brain edema following TBI (100). Recently, an exosome study found that miR-873a-5p, one of the astrocyte-derived exosomes’ critical components, can attenuate the microglia-mediated neuroinflammation by inhibiting the NF-κB signaling pathway (101).

Indeed, inhibiting microglia activation can reduce white matter injury (WMI) because the preservation of myelin is related to M2 microglial polarization switching modulation *via* the PTEN/PI3K/Akt signaling pathway (102). Emerging evidence shows that the protein kinase R-like endoplasmic reticulum kinase (PERK) in neurons will upstream M1 microglial polarization and increase IFN-β releasing, which is followed by Th1 cell infiltration, eventually causes the decline of oligodendrocyte precursor or mature oligodendrocytes cells (103). More recently, the PERK signaling-mediated crosstalk between microglia and oligodendrocytes has been highlighted again as it was consistent with the finding that WMI or oligodendrocyte loss is favored by the pro-inflammatory environment (104).

There are still lacking direct communication theories between astrocytes and oligodendrocytes in neuroinflammation following TBI. Interestingly, it has been found that IL-33, mainly produced by astrocytes and oligodendrocytes, will be elevated to the maximum after 72 h, and it promotes the recruitment of microglia/macrophages following TBI (105). Moreover, astrocytes and oligodendrocytes can communicate through connexins (Cx), in that the astrocytic Cx43 joined with the oligodendrocytic Cx47 regulates both myelination and demyelination (106). Recently, a study has found that the upregulated functional astrocytic Cx43 expression will promote the mitochondria transmission from astrocytes to neurons, which might benefit the protection of neurons after TBI (107). Thus, it remains to be determined experimentally whether the Cx43 can be a bridge molecule to untangle the complex relationship between astrocytes and oligodendrocytes in TBI-related neuroinflammation.

Moreover, beyond that, the impact of neuron-glial antigen 2 (NG2) glia on microglial activation has been confirmed experimentally, of which NG2 glia regulates the neuroinflammatory response *via* the TGF-β2 axis (108). It has also been found that the lack of NG2 exacerbates neurological deterioration through the abnormal activation of microglia or astrocytes and increased peripheral immune cell recruitment to the injured brain (109). At this point, NG2 glia may counteract adverse glial responses and thus achieve neurological function repair in TBI. Indeed, the contribution of NG2 glia to neuroinflammation and their communications to the other glia following TBI and the molecular players (transcripts and proteins) that differentiate NG2 cells in this scenario remains an open question.

**Figure 1:**
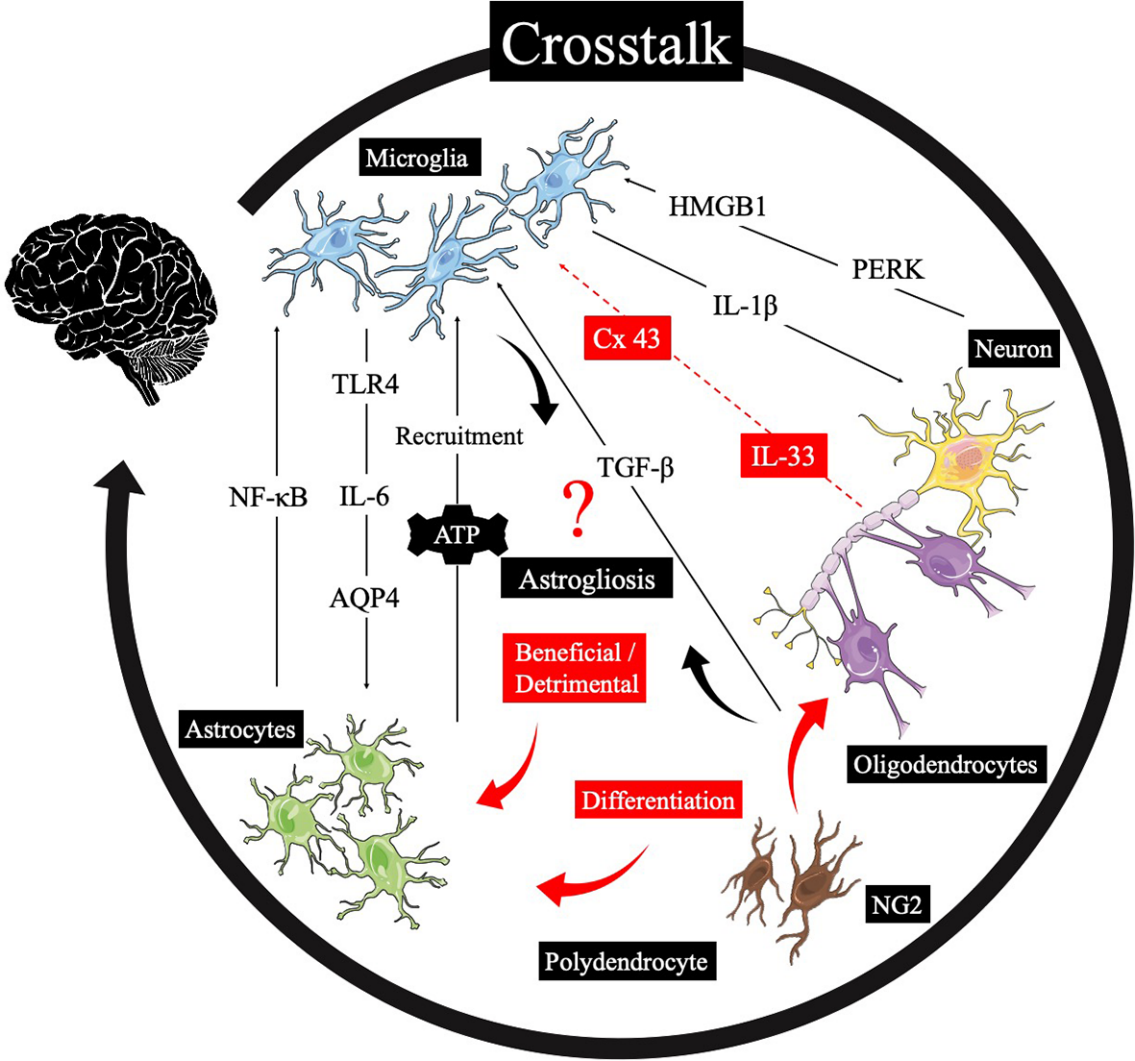
The crosstalk of cells in the neuroinflammation. After TBI, the microglia can interact with astrocytes, the oligodendrocyte, neurons, and NG2 cells through several pathways, and the “closed-loop” mechanisms of their communications remain unclear. In addition, the differentiation between the NG2 cells and polydendrocytes or oligodendrocytes is still not clear, and their difference might play a role in neuroinflammation. (black color: settled cell–cell crosstalk mechanisms, red color: possible crosstalk mechanisms need to be certified).

The intercellular crosstalk between astrocytes and microglia has contributed an essential role to the neuroinflammation following TBI (65). To date, several signal pathways were found to participate in the crosstalk between the microglia and the astrocytes, oligodendrocyte, neurons, and NG2 cells (**Figure 1**). Finding the integration and coordination of the glial responses to TBI is a broad field worthy of exploration.

## Difference in the Diffuse Axonal Injury

**Figure 2:**
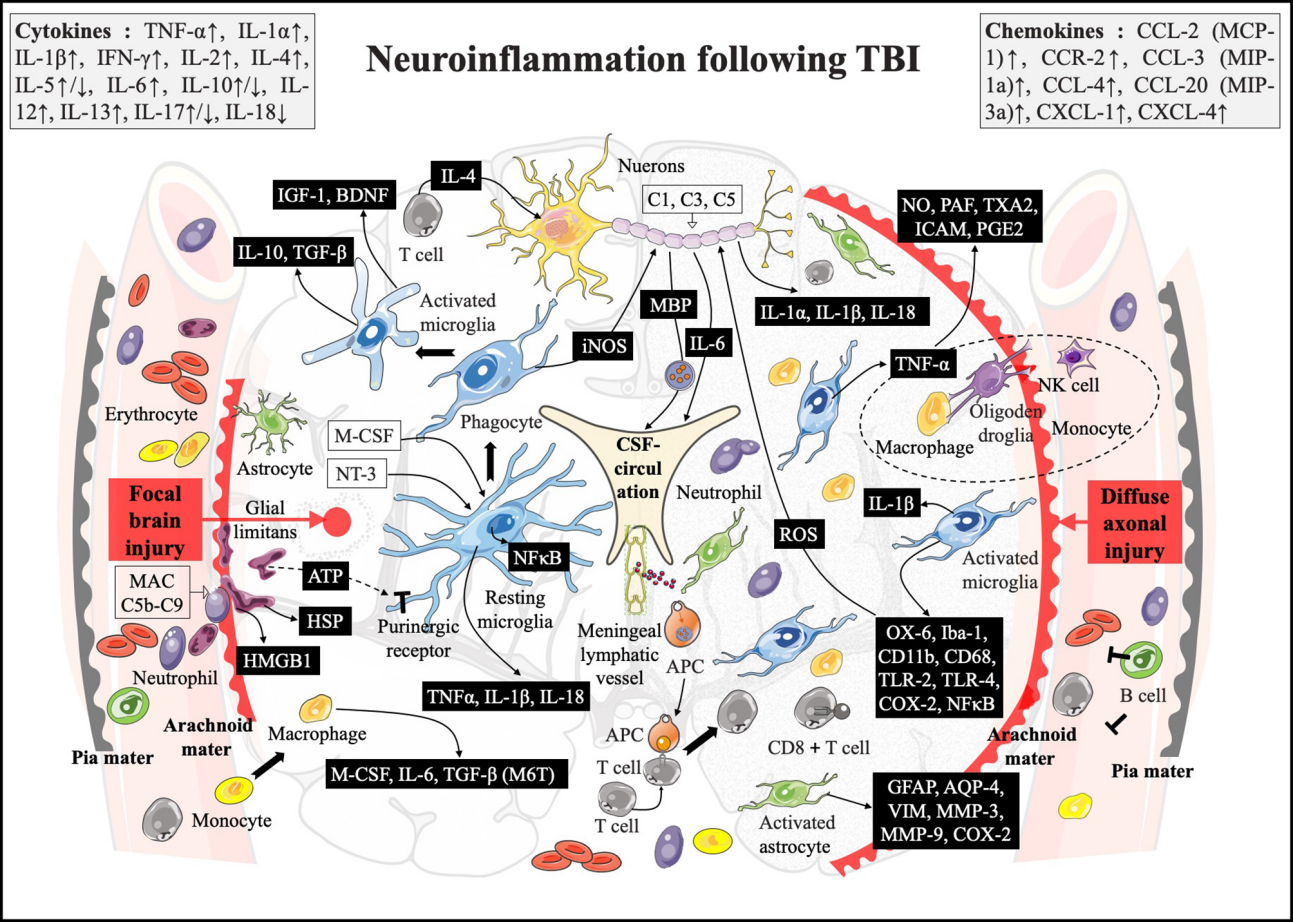
A brief comparison of the neuroinflammation in two main subtypes of TBI. In the FBI (the left side), it begins with the complement activation and the initiators (including HMGB1, HSP, and ATP) releasing from the damaged meninges and parenchyma within minutes following brain injury. Those compliments will bind to DAMP sensors such as purinergic receptors and TLRs that immediately induce resident microglia activation followed by inflammasome assembling. Then, a variable number of cytokines and chemokines generating and the NF-κB translocating into the nuclei induce an immunological reprogramming. Meanwhile, it can also induce the recruitment of neutrophils and macrophages to the injured meninges and/or perivascular regions, by which more chemokines and cytokines proliferate and the inflammatory response amplifies. In the DAI (the right side), in addition to the above-mentioned processes, the MBP is released from the damaged neural myelin involved in CSF circulation. T cells can be recruited to the damage site *via* a damaged BBB and/or meningeal lymphatic vessel, and the local APCs subsequently present it. Moreover, massively activated astrocytes and oligodendrocytes and their crosstalk can participate in immunogenic signal activation, which takes precedence over peripheral immune infiltration (such as neutrophils and macrophages).

As discussed previously in this review, the inflammatory response in FBI is characterized by the microglial activation and leukocyte infiltration around the parenchymal contusion. In contrast to the FBI, the damage of DAI scattered throughout the subcortical WM, including the corpus callosum, thalamus, and brain stem (110). To our knowledge, when TBI damages gray matter (GM) and WM, astrocytes proliferate preferentially in GM, while microglia are more abundant in WM, and the WM lesion will exert a strong influence on the GM glial cell proliferation at early 3 days post-injury (111). The difference of the inflammatory response between FBI and DAI should be considered carefully because widespread meningeal injury and extensive WMI often appears in it **(** **Figure 2** **)**.

Increasing evidence has revealed the difference in cellular immune response, cytokine activation, and chemokines in DAI. It has been found that the microglia that express the lectin galectin-3/Mac-2 will last up to 28 days and were most evident at 24 h post-injury (112). The activated microglia are located mainly in the hippocampus and cortex as early as 4 h and MHC-II upregulation in WM tracts at 24 h following injury. It is noteworthy that the meningeal and perivascular macrophage infiltration had marginal influence from 24–48 h up to 2 weeks. Moreover, a little parenchymal infiltration of the lymphocytes and granulocytes was observed throughout the brain, which means a small contribution of those two populations of immune cells in the neuroinflammation of DAI (110). In addition to focusing on the number of activated microglial cells and macrophages, electron microscopy findings suggest that they exhibit consistent immune cell interactions for more than 1 week. Although precise interactions remain uncertain, spatiotemporal microstructural immune cell alterations will be a focus of the follow-up studies (113).

Meanwhile, it has been reported that the high expression of IL-1β was strongly correlated to the DAI (114), and the inflammation of the levels of TNF-α and Il-1β is dynamically controlled by macrophages (115). Previous evidence suggested that neuroinflammation was enhanced with the increase of IL-1β, IL-6, and TNF-ɑ (116). Despite the upregulation of ICAM-1 in the cortex, thalamus, and corpus callosum, there was no infiltration of neutrophils detected in DAI. Moreover, MCP-1 (a recruitment factor of borne monocytes) rather than MIP-2 (a chemotactic factor for neutrophils) was significantly elevated after DAI. These findings imply that a massive immunogenic signal activation always takes precedence over the peripheral immune infiltration in DAI. Indeed, a recent study found that there was a significant increase for five chemokines (CCL11, CX3CL1, CXCL5, CCL2, CCL3), ten cytokines (IL-1α, IL-1β, IL-4, IL-6, IL-10, IL-13, IL-17α, IL-18, IFN-γ, TNF-α), and four growth factors [EGF, GM-CSF, leptin, vascular endothelial growth factor (VEGF)] following DAI (117). At this point, DAI could induce more inflammatory biomarker activation than FBI.

Accordingly, a more significant glial aviation involved in cytokine/chemokine release and cytokine-mediated signaling was found more complicated in the neuroinflammation of DAI than FBI (110). It is reasonable to believe that using FBI models to generalize the general inflammatory response process in DAI often risks conclusions inappropriately. For example, the M2 microglia phenotype was previously known to be associated with anti-inflammatory and neurogenesis effects in the FBI. However, neuroinflammation persists without improvement despite the acute enrichment of the M2 microglial phenotype in DAI (118).

## The Regularity of Neuroinflammation Following TBI

**Figure 3:**
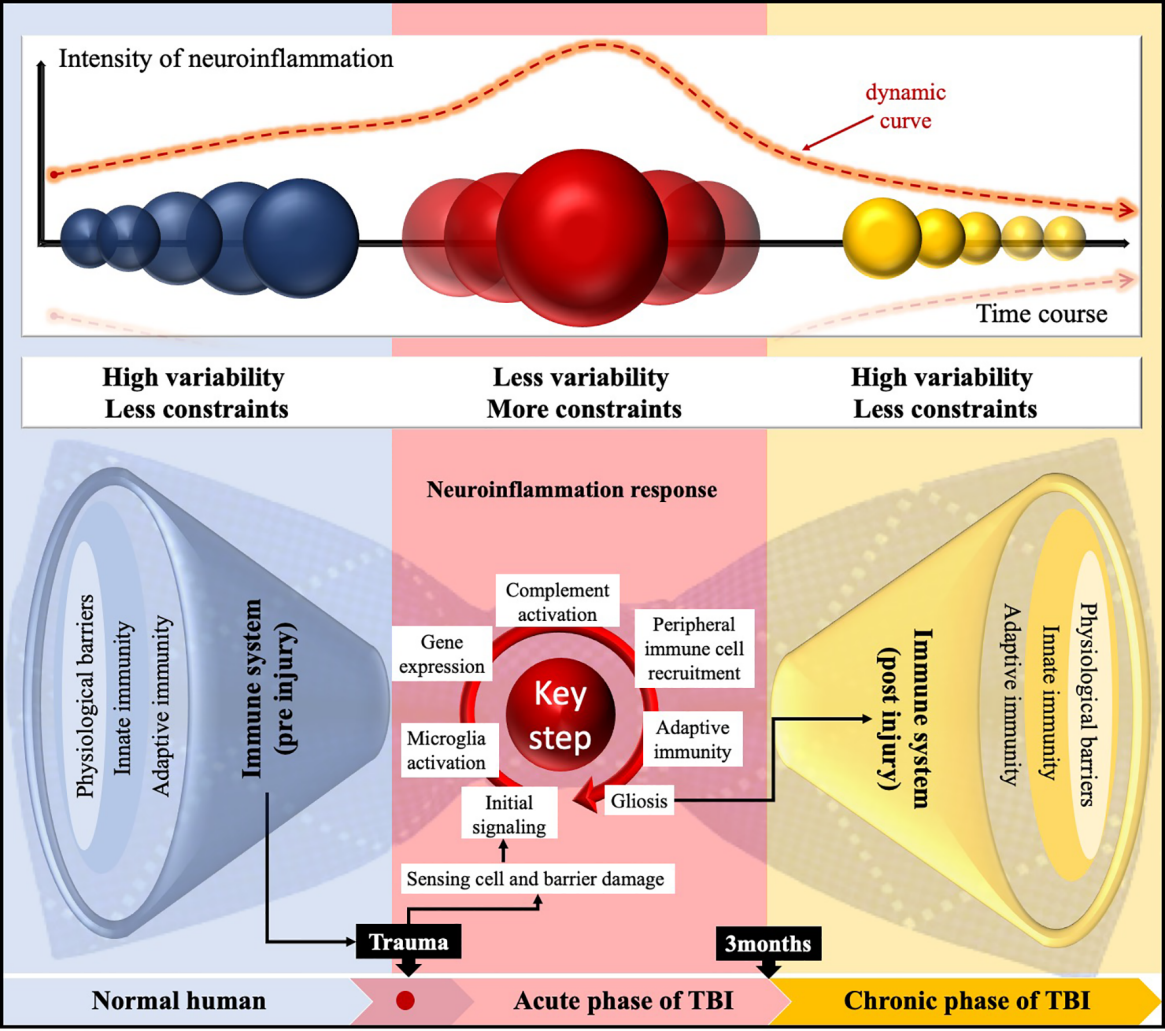
Schematic representation of the time-related paradigm shift in neuroinflammation following TBI employing a bow-tie paradigm. The characteristic of immunogenicity in the CNS was high variability and fewer constraints pre-TBI. Then, in the core, the neuroinflammation is “compressed” by relatively regular rules or steps and processed into chronic neuroinflammation following TBI. Notably, there was a dynamic course of the functional diversity; temporal intensity of response; and dynamic changes in inflammatory gene expression, cell activation, and their interaction network.

Although the role of innate and adaptive immunity in CNS has not been entirely clear, TBI is the leading risk factor that destroys the homeostasis in it. Neuroinflammation following TBI has now been recognized as a complex interaction between central and peripheral soluble components, influenced by the baseline characteristics (age, sex), types of injury (focal, diffuse), degrees of injury (mild, moderate, severe), secondary insult, genetic variability, and therapeutic interventions (6). The regularity of this process includes the following steps: initial signaling, resident microglial activation, gene expression, complement activation, peripheral immune cell recruitment, and adaptive immunity. To our knowledge, bow-tie is a general term that refers to an ordered and recurrent system underlying the complexity of technological networks (119). With this in mind, we figuratively present a panoramic paradigm of the pre- and post-TBI inflammation with the help of the bow-tie characteristic (**Figure 3**). Significantly, TBI could accelerate immune aging, displaying chronic deficits that affect the long-term consequences of systemic immune function (120). Therefore, there are reasons to believe that the persistent neuroinflammation follow-up TBI leads to several neurological disorders, including epilepsy, neurodegenerative disorders, chronic traumatic encephalopathy, and Alzheimer’s disease (121).

## Perspective

Post-TBI inflammation can be beneficial by promoting both the clearances of debris and neuron regeneration and/or detrimental because it mediates neuronal death and chronic neurodegeneration. Applying the graph theory to interpret the complexity of neuroinflammation following TBI is a crossproductof a multidisciplinary approach. After TBI, the activity of microglia and infiltrating macrophages is crucial to the majority of neuroinflammation, and glial interaction signals might represent the new target of research on the mechanism of neuroimmune response after TBI. Future studies on neuroinflammation should specify the patient characteristics, injury type and severity, dynamics in neuroinflammation, and even immune cell heterogeneity. In addition, an individualized design of immune tolerance therapy is required because targeting the same inflammatory pathways in different types of TBI might turn over the anticipated neurological effects in immunotherapy. A possible hypothesis is that the collective signaling pathways that generate classically and alternatively activated neuroinflammation may be related to TBI-induced injury. It is a fundamental principle to study neuroinflammation and every kind of pathway and the relationship between them. All in all, our bow-tie framework can help set goals for different stages of immunotherapy in TBI patients: for the acute phase, reverse neuroinflammation to achieve neuroprotection; for the chronic phase, stop persistent neuroinflammation.

## Author Contributions

Z-xQ, Z-yX, and ZW contributed to literature extraction and prepared the manuscript. R-zZ and K-yL drafted this manuscript. X-hW and YM supervised the study. All authors read and approved the final manuscript.

## Funding

This work was supported by the Shanghai Municipal Science and Technology Major Project (No. 2018SHZDZX01), ZJ Lab, and Shanghai Center for Brain Science and Brain-Inspired Technology, and the National major pre-research project (pilot project) (No. IDF151042).

## Conflict of Interest

The authors declare that the research was conducted in the absence of any commercial or financial relationships that could be construed as a potential conflict of interest.

## Publisher’s Note

All claims expressed in this article are solely those of the authors and do not necessarily represent those of their affiliated organizations, or those of the publisher, the editors and the reviewers. Any product that may be evaluated in this article, or claim that may be made by its manufacturer, is not guaranteed or endorsed by the publisher.
